# Microwave-Assisted Subcritical Water Extraction of Hemp Seeds for the Simultaneous Recovery of Proteins and Phenolic Compounds

**DOI:** 10.3390/foods14244201

**Published:** 2025-12-07

**Authors:** Aziadé Chemat, Salah Chaji, Christian Cravotto, Giorgio Capaldi, Luisa Boffa, Giorgio Grillo, Anne-Sylvie Fabiano-Tixier, Giancarlo Cravotto

**Affiliations:** 1 Department of Drug Science and Technology, University of Turin, 10125 Turin, Italy; aziade.chemat@gmail.com (A.C.); salah.chaji@unito.it (S.C.); giorgio.capaldi@unito.it (G.C.); luisa.boffa@unito.it (L.B.); giorgio.grillo@unito.it (G.G.); 2 GREEN Extraction Team, National Research Institute for Agriculture, Food and Environment (INRAE), Mixed Research Unit (UMR) 408, Avignon University, 84000 Avignon, France; 3 Unité de Recherche et de Développement (URD) Agro-Biotechnologies Industrielles (ABI), Centre Européen de Biotechnologie et de Bioéconomie (CEBB), AgroParisTech, 51110 Pomacle, France; christian.cravotto@agroparistech.fr

**Keywords:** hemp seeds, *Cannabis sativa* L., subcritical water extraction, proteins, polyphenols, biorefinery

## Abstract

This work presents an innovative microwave-assisted subcritical water extraction (MA-SWE) approach for the simultaneous recovery of proteins and phenolic compounds from hemp seeds (HS). The extraction temperature (100–180 °C), time (5–60 min), and solid-to-liquid ratio (1:5–1:30 *w*/*v*) were optimized via Box–Behnken design. The effects of whole, crushed and defatted HS were investigated, with defatted HS exhibiting higher selectivity for proteins and polyphenols. Two optimization strategies were explored: one maximizing protein and polyphenol yields and another minimizing solvent and energy consumption, while maintaining competitive yields. The maximized conditions (MAPPY) were 180 °C, 57 min, and a ratio of 1:28, providing a protein selectivity of 48.91 g/100 g of dry extract (DE) and a total phenolic content of 7.24 g gallic acid equivalent/100 g DE. Regarding functional properties, both strategies yielded extracts with similar performance; however, the maximization strategy resulted in higher emulsifying capacities. These results support the industrial valorization of defatted HS by MA-SWE under optimized conditions to produce antioxidant- and protein-rich ingredients.

## 1. Introduction

Agrifood industries are broadening their horizons towards more sustainable production models; a transition that is being driven by the integration of environmentally friendly and innovative technologies [1]. This approach will ensure compliance with international and national legislation concerning the management of consumption systems. In addition, it will establish new opportunities for industrial actors through the valorization of by-products generated during the production system. This, in turn, will enhance the economic income of their value chains. Furthermore, the willingness of consumers to pay a premium for bio-based products, including plant-based nutraceutical supplements and bio-based health-promoting products, provides an additional boost to industry actors, stakeholders and policymakers, encouraging them to accelerate the establishment of innovative valorization strategies [2].

*Cannabis sativa* L. is widely recognized for its psychoactive properties, which are primarily linked to the cannabinoid delta-9-tetrahydrocannabinol (Δ^9^-THC) [3]. Beyond this, it offers a vast array of extractable primary and secondary metabolites, showcasing its potential for innovative valorization strategies. Indeed, *Cannabis* comprises a diverse range of species, including hemp varieties, which exhibit minimal levels of Δ^9^-THC and are renowned for their utilization as a fiber crop, from which textiles and paper are derived [4,5]. Additionally, hemp can also be used as an ingredient in the formulation of food and pharmaceutical products [6,7,8]. The extensive cultivation of hemp can be attributed to the fact that all parts of the plant have a practical application, making it an intriguing and economically viable option for farmers. Indeed, a multitude of purposes have been identified for the plant’s components, including its fiber, hurds and flowers as well as its leaves, roots, sprouts and seeds [8]. In accordance with applicable legislation, the designation and utilization of hemp is permitted when the concentration of Δ^9^-THC in the dried material does not exceed 0.2% [6].

Hemp seeds (HS) oil is among the most valuable hemp components, comprising between 19 and 38% of the seed’s total weight [7]. The lipidic portion is primarily composed of polyunsaturated acids, with linoleic acid (omega-6) representing approximately 60% and α-linolenic acid (omega-3) accounting for approximately 20% [9,10]. This ratio of omega-6 to omega-3 is regarded as optimal for human health [8]. Additionally, the high nutritional profile of HS is mainly attributed to its proteome, as it contains around 181 expressed proteins [11]. Hemp proteins are primarily composed of edestin (57–76%), albumin (15–24%), and vicilin (9–19%), as evidenced by previous studies [12,13]. The isolated peptides have exhibited noteworthy antioxidant properties, as demonstrated by Leonard et al. (2020) [14]. The amino acids have been identified, by House and colleagues (2010), as being highly digestible [15], and edestin has been associated with the promotion of a healthy immune system and the alleviation of stress [3]. The peptide components have demonstrated anti-hypertensive and cardioprotective effects [14]. Additionally, arginine has been linked to improved cardiovascular health [16]. Despite being the least studied components of HS, some papers have indicated the presence of several phenolic compounds, such as caffeic acid, quercetin, luteolin, gallic acid, (+)-catechin, syringic acid, myricetin and 4-hydroxybenzoic acid [17,18]. The phenolic fraction constitutes a minor component of HS, accounting for approximately 0.4% of the whole seed and 0.2% of the oil, expressed as gallic acid equivalents (GAE) [19].

HS are primarily extracted for their oil, proteins and polyphenols, with cold pressing and hexane extraction being the most common methods for recovery of oil. Cold pressing is a mechanical extraction technique that can yield between 23.60 and 27.97% of oil [20]. In a study conducted by Devi et al. (2019), high oil yields were achieved using hexane together with various extraction techniques, including Soxhlet (36.3%), ultrasound (35.72%), and a combination of Soxhlet and ultrasound pretreatment (37.30%, 36.94%, respectively) [21]. The same study compared its findings to those of supercritical fluid extraction (SFE) using CO_2_ and ethanol as a co-solvent, which yielded 36.26% oil. Considering the high cost of SFE, the use of an environmentally friendly solvent, such as 2-methyloxolane (2-MeOx), which has shown promising results, offers a viable alternative to hexane. Most importantly, industrial plants designed for hexane can be easily adapted for 2-MeOx with only minor modifications [22].

Proteins are commonly extracted from hemp cakes and meal using the pH-shift method [12,23]. This technique relies on a prolonged stirring period and the application of high centrifugation forces ranging between 8000 and 12,000× *g*. The resulting protein isolates contain between 60 and 70% proteins, as reported by Potin et al. (2019) [23], and a concentration above 96% in the case of research conducted by Liu et al. (2023) [12].

In the extraction of polyphenols from HS cake, dynamic maceration with a solvent mixture of methanol–acetone–water (7:7:6, *v*/*v*/*v*) gave better results than the use of methanol, ethanol, hexane and acetone [24]. Another relevant study demonstrated that total phenolic content (TPC) was enhanced when innovative techniques were applied; namely, microwave-assisted extraction (MAE) combined with pulsed electrical field (PEF), and MAE combined with ultrasonication (US) [25]. Studies conducted by Švarc-Gajić et al. [17,26] have explored the potential of subcritical water extraction (SWE), using a home-built machine. Interesting results were achieved when a variety of parameters were examined, including atmospheres with nitrogen, nitrogen with acidified water, and carbon dioxide.

SWE is, indeed, a promising, environmentally friendly method for the extraction of a wide polarity range of compounds (e.g., polyphenols, proteins, peptides) by adjusting extraction temperature and pressure conditions [27,28]. In SWE, water is kept in a liquid state under sufficient pressure at temperatures between its boiling point (100 °C) and its critical point (374 °C). However, water’s polarity changes from strongly polar to much less polar depending on the temperature and pressure, enabling the extraction of various types of compounds [28]. The high temperatures improve the penetration of the solvent, while reducing the viscosity and accelerating the dissolution of extractable compounds [29]. However, excessively high temperatures can degrade sensitive compounds, with phenols being particularly sensitive, even after brief exposure [30,31]. Similarly, elevated temperatures can cause protein degradation [31]. The degradation temperatures of “unstable” compounds vary depending on the biomass and the specific compounds involved [28,30]. However, due to the wide polarity changes in water, SWE can enable the simultaneous recovery of protein and phenolic fractions in a single-step operation; a distinct advantage over sequential, organic solvent-intensive conventional methods. Furthermore, when using microwave irradiation in the SWE process, the heat and mass transfer can be enhanced since the microwave energy is directly delivered to the raw matrix, unlike conventional heating, where the energy is transferred by convection, conduction, and radiation phenomena [32]. Microwave-assisted SWE (MA-SWE) is also linked to lower energy consumption compared to conventional heating SWE, owing to its rapid volumetric heating, reduced thermal losses, and shorter extraction times [33].

For this, SWE stands out as a particularly relevant technology for reaching this integrated biorefinery goal. Furthermore, from an environmental point of view, the use of SWE was found to achieve a better environmental score compared to conventional methods such as reflux-based extraction, according to green metrics including Green Motion, Reaction Mass Efficiency, and E-Factor, as recently reported by our research group [34].

This study focuses on HS valorization through the simultaneous recovery of both nutritional and health-promoting compounds in an effective and non-selective 1-step extraction. To the best of our knowledge, this is the first study to investigate the potential use of SWE for the simultaneous recovery of both protein and phenolic compounds. The aim is to develop a MA-SWE-based approach combined with defatting to obtain protein- and polyphenol-rich extracts. To this end, the impact of matrix defatting prior to MA-SWE has been investigated using both defatted and non-defatted seeds, while a Box–Behnken optimization design was employed to identify optimal extraction conditions.

## 2. Materials and Methods

### 2.1. Biomass

HS were provided by the company Molino Crisafulli (Sicily, Italy). The material was stored in a sealed glass container at room temperature. The seeds were used in three different states: whole (WO); crushed by using a roller flaker (CR) (particle size < 0.5 cm); and finally defatted (DF), which the making is described in Section 2.4.3.

### 2.2. Microwave-Assisted Subcritical Water Extraction

MA-SWE was conducted using a multimodal microwave reactor (Synthwave ©, Milestone, Bergamo, Italy). The system allows configurable parameters, including extraction temperature, heating ramp time, extraction time, pressure and microwave power. Before each test, the reactor was purged three times with N_2_ to remove oxygen from the system and minimize oxidative stress. The initially loaded pressure was set to 9 bars. During extraction, the pressure increased as the temperature rose to the target extraction temperature, reaching 16 bars within a 5 min ramp, and subsequently decreased once heating was stopped. The microwave power, used as a heating system, was automatically regulated by the instrument to maintain a set processing temperature, using an irradiation power of 1500 W.

Prior to the method-optimization process, a single protocol was performed to reveal the potential effect of HS pretreatment on extraction efficiency. The experimental conditions used were as follows: a temperature heating ramp of 5 min was set to reach the extraction temperature of 150 °C, and once this temperature was achieved, extraction was carried out for 30 min. These conditions were selected so that the fixed temperature and time parameters are not excessively elevated to the extent that they may lead to degradation in the components of interest, while being, nevertheless, sufficiently robust to drive the extraction process [28].

The experiments were conducted with a matrix quantity varying between 0.5 and 3 g. Different volumes were used in the extraction cell depending on the experimental conditions during the optimization process, with the maximum volume applied being 600 mL. After extraction, the extracts obtained were centrifuged (Cence Hunan Xiangyi Laboratory Instrument Development Co., Ltd., Xiangyi, China) at 4200 rpm for 10 min, and the supernatant was filtered under vacuum. The dry extracts were finally recovered after freeze drying (LyoQuest-85, Telstar, Madrid, Spain), weighed and stored under dark conditions at 4 °C for further analysis. The extraction yields were expressed as percentages of dry extract over the raw matrix (*w*/*w*).

### 2.3. Optimization—Experimental Design

An experimental design procedure was performed to determine the optimal conditions for the extraction process, with the objective of establishing two distinct optimal sets of parameters; one, named “Maximum Achieved Protein and Polyphenol Yield” (MAPPY), referring to the highest yield, and the other, named “Optimal Economic Conditions” (OEC), to the most favorable experimental conditions for achieving good yields while simultaneously minimizing energy consumption.

A Box–Behnken procedure was performed using Design-Expert version 13 (Stat-Ease, Inc., Minneapolis, MN, USA). A total of 16 experiments were carried out using 4 central points. Three variables were investigated at levels 1, 0, −1; temperature, ranging from 100 to 180 °C, time, from 5 to 60 min, and the solid-to-liquid ratio, which fluctuated between 1:5 and 1:30 (*w*/*v*). The experimental and coded values are reported in Table 1.

The responses studied were extraction yield (extract/100 g dry matter (DM)), protein selectivity (g proteins/100 g extract), protein recovery (%) and TPC (g GAE/100 g extract). A quadratic equation was used (Equation (1)) for this experimental design:

(1) $$y=\beta_{0}+\sum_{i=1}^{3}\beta_{i}X_{i}+\sum_{i=1}^{3}{\beta_{ii}X}_{i}^{2}+\sum_{i<j=1}^{3}\beta_{ij}X_{i}X_{j}$$

where y is the estimated response and expressions β_0_, β_i_, β_ii_ and β_ij_ are the equation constants (y-intercept) and the regression coefficients for linear, quadratic and interaction terms, respectively. The results were statistically tested using analysis of variance (ANOVA). The adequacy of the models was evaluated using the coefficient of determination (R^2^), the coefficient of variance (CV) and the *p*-value for the model and lack-of-fit testing.

### 2.4. Analytical Methods

#### 2.4.1. Polyphenols Content

First, total phenolic content (TPC) was determined in the raw material. For this task, three consecutive reflux extractions were performed with a hydroalcoholic mixture (70:30 water–ethanol, *v*/*v*) at a 1:30 (*w*/*v*) biomass-to-solvent ratio, for 1 h. After each cycle, the extract was separated, and the residual biomass was dried and re-extracted under the same conditions. The pooled extracts were freeze-dried and analyzed for their TPCs.

The Folin–Ciocalteu method was employed to determine the TPCs of the extracts, according to the method reported by Hillis and Swain (1959) [35]. Following a dilution of the sample, 250 μL was combined with 4 mL of distilled water, 500 μL of sodium bicarbonate (10% *w*/*v*) and 250 μL of Folin–Ciocalteu reagent (diluted 1:1 with distilled water). Subsequently, the mixture was incubated in the dark for 25 min and the absorbance was measured at a wavelength of 725 nm using an ultraviolet-visible (UV-visible) spectrophotometer (Cary 60, Agilent Technologies, Santa Clara, CA, USA). The calibration curve used for the Folin–Ciocalteu phenolic assay is reported in Figure S1. All results are expressed as an average of a triplicate, in gallic acid equivalent (GAE).

#### 2.4.2. Protein Content

The protein content of the raw matrix and extracts was quantified using the Kjeldahl method, according to the AFNOR T90-110 standard [36], using a DK 6 Kjeldahl Digestion unit (Velp Scientifica, Usmate Velate, Italy), with an EasyKjel distillation apparatus (Buchi, Flawil, Switzerland). The protein content was determined using the conversion factor 6.25.

#### 2.4.3. Oil Content

Crushed seeds were extracted via dynamic maceration (at 400 rpm) with hexane at ambient temperature using a solid-to-liquid (S/L) ratio of 1:20 (*w*/*v*). The extraction process was repeated three times, with each extraction lasting for 1 h. Subsequently, the extract was filtered on a Buchner funnel with hexane and was distilled under vacuum. The material was then spread over aluminum foil and left under a hood overnight to allow the hexane to evaporate. The oil was weighed and expressed as a percentage. The obtained defatted seeds were then used for extraction.

#### 2.4.4. Humidity and Ash Content

Seed humidity and ash content were determined in a two-step process. An initial quantity of 1 g was weighed and placed in a crucible. Initially, the seeds were placed in an oven at 105 °C for a period of 16 h. Following this, the difference in weight before (W_i_) and after (W_f_) oven drying gives the moisture content of the sample, as shown in Equation (2):

(2) $$Moisture\%=\;\frac{\left(W_{i}\;-W_{f}\right)}{W_{i}}\times 100$$

Subsequently, the crucible was placed in a muffle furnace, which performed a temperature ramp to 650 °C, over a 4 h period. Afterwards, the crucible was left in a desiccator to cool for 1 h and then weighed. The total ash content was calculated as highlighted in the following Equation (3):

(3) $$Ash\;content\;\left(\%\right)=\frac{\left(W_{f}-W_{i}\right)}{W_{s}}\times 100$$

where W_f_ is the final gross weight of the crucible, W_i_ is the initial gross weight, and W_s_ is the amount of sample inserted into the furnace.

### 2.5. Functional Properties

The extracts obtained according to the two methods, MAPPY and OEC, were analyzed for their functional properties, including oil holding capacity (OHC), foam capacity (FC), foam stability (FS), emulsifying activity (EA) and emulsion stability. The water holding capacity of the extracts could not be determined since both extracts were soluble in water and did not form any insoluble residue able to retain water during the measurement. All results were expressed as average of a triplicate ± standard deviation.

#### 2.5.1. Oil Holding Capacity

0.5 g of the extract was mixed with 7.5 mL of soybean oil, vortexed (MIX ARGOLab, Turin, Italy) for 1 min, rested for 30 min, then centrifuged at 4200 rpm for 15 min. After removing the supernatant, the tube was weighed. OHC was calculated based on weight differences and expressed as percentage of oil retained relative to the dry extract (DE) [37].

#### 2.5.2. Foaming Properties

Both FC and FS were determined following the methodology described by Vinayashree et al. (2021) with slight modifications [38]. A 10 mL sample of protein solution of a concentration of 10 mg/mL was homogenized for 5 min. Afterwards, the solution was transferred to a graduated cylinder, and the initial and final volumes were noted. FC was defined as the percentage increase in volume relative to the initial volume, while FS was assessed by monitoring the volumetric changes over 2 h of storage. Calculations of FC and FS were determined using the following equations:

(4) $$FC\%=\frac{volume\;after\;stirring-volume\;before\;stirring}{volume\;before\;stirring}\times 100$$

(5) $$FS\%=\frac{volume\;after\;120\;\min\;of\;standing-volume\;before\;stirring}{volume\;after\;stirring-volume\;before\;stirring}\times 100$$

#### 2.5.3. Emulsification Properties

A turbidimetric method was employed to determine EA and ES as described by Horax et al. (2011) [39]. Briefly, sunflower oil was added to protein solution (0.5%) at a ratio of 1:3 (*v*/*v*), and the mixture was homogenized for 1 min, using a homogenizer (IKA T50, ULTRA-TURRAX, Staufen, Germany) to form an emulsion. Subsequently, 50 µL of the emulsion were transferred to a test tube containing 5 mL of 0.1% sodium dodecyl sulfate (SDS) (*w*/*v*) at 0 and 10 min after homogenization. The absorbance of the solutions was measured at 500 nm using a spectrophotometer (Cary 60, Agilent Technologies, Santa Clara, CA, USA). EA was expressed as the absorbance at 0 min, while ES was calculated by the following equation: ES = T_0_ × Δt/ΔT; where ΔT was the change in absorbance occurred in a period of 10 min (Δt).

### 2.6. Statistical Analysis

The results obtained in this study are reported as mean values of triplicates ± standard deviation (SD). One-way analysis of variance (ANOVA) was performed, and mean values were compared by Tukey’s test at 5% of probability of error. All statistical analyses were performed using IBM SPSS Statistics software (SPSS for Windows, Version 20, SPSS Inc., Chicago, IL, USA).

## 3. Results and Discussion

### 3.1. Biomass Characterization

Raw HS had a moisture content of 6.5% and an ash content of 5.2%. This is in accordance with findings reported by Callaway (2004) [40]. The biomass exhibited 21.60% protein, 3.83% total phenolic content and 31.44% oil. After defatting, the protein content increased to 29.49%, and the phenolic content rose to 5.85%.

A review of the literature highlights significant variability in HS composition, with protein content ranging from 20 to 38% [10,16], phenolic content from 0.08 to 5.16% [19], and oil content from 19 to 38% [9,41]. These variations are influenced by factors such as cultivar, environmental conditions, and agronomical practices [42,43].

### 3.2. Impact of Matrix Pretreatment on MA-SWE

**Figure 1:**
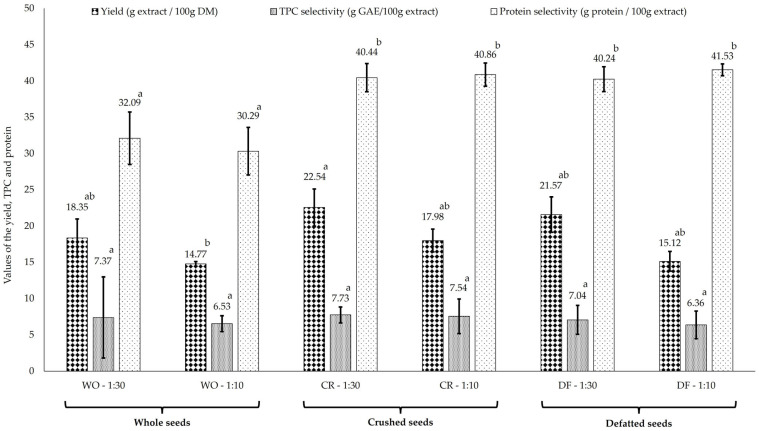
Impact of matrix pretreatment on yield (g extract/100 g DM), polyphenols (g GAE/100 g extract), and proteins (g proteins/100 g extract) with MA-SWE at 1:10 and 1:30 ratios. Distinct lowercase letters represent significant differences (*p* < 0.05).

HS are primarily extracted for their oil, before further steps for the recovery of other valuable compounds. Concurrent research efforts have focused on the extraction of nutritional and health-promoting compounds, including proteins and polyphenols, mainly from defatted seeds, which are also known as cakes after mechanical pressing or meals when the matrix is defatted with solvents [44]. Herein, we have investigated the effect of different HS pretreatments on the MA-SWE-recovery process. The extraction and comparison of whole (WO), crushed (CR) and defatted (DF) HS at ratios of 1:10 and 1:30 were conducted. The extraction yield, protein selectivity and polyphenol selectivity were the parameters of comparison. The results are presented in Figure 1.

As can be observed in Figure 1, a higher ratio of 1:30 gave better results in terms of yield for all the samples studied. However, no significant differences in protein selectivity and TPC were observed between the ratios for each type of pretreatment (*p* > 0.05). Bearing in mind the need for minimizing water consumption for sustainability purposes, this suggests that the lower ratio seems to be more suitable for achieving good selectivity towards proteins and polyphenols.

The WO-HS gave lower results in terms of yield and proteins selectivity. The highest yield was obtained from CR-HS with a ratio of 1:30 (22.54 g extract/100 g DM) while the lowest was achieved in WO-HS with a ratio of 1:10 (14.77 g extract/100 g DM) (*p*-value < 0.05). The observed trend can be attributed to the high contact surface of the matrix with the solvent at higher ratios and when crushing the seeds, thereby favoring the extraction of various compounds as a response to their affinity with the solvent. Furthermore, the higher yields obtained from crushed seeds compared to crushed and defatted seeds may be attributed to several factors. For instance, the defatting process can induce structural modifications within the seed matrix, including protein structural alterations, which in turn reduce the accessibility and solubility of proteins [45]. A fraction of the phenolic compounds may also be removed during defatting, depending on their affinity for the solvent used, thereby reducing their content in the final extract [46]. The highest protein selectivity was achieved in DF-HS extracted with a ratio of 1:10 (41.53 g proteins/100 g extract), while the lowest was linked to the samples of the WO-HS extracted with a ratio of 1:10 (30.29 g proteins/100 g extract) (*p*-value < 0.05). The main factor contributing to this phenomenon is the elimination of the lipid fraction, which in turn leads to an increase in the content of other compounds, including proteins, in the final extract.

Turning to focus on TPC values, no significant differences were noticed in the three types of samples, with the highest value being reached in the CR-HS samples extracted at a ratio of 1:30 (7.73 g GAE/100 g extract). This can be attributed to the possible extraction of some polyphenols by hexane during the defatting step, which, in turn, limits its level in the final extract from defatted seeds [22]. However, in a study reported by Menga et al., changes in the concentrations of individual phenolic compounds in defatted and whole seeds were observed to exhibit differing trends according to the type of compound, the cultivar and the crop year [47]. The comparable values obtained for WO and CR seeds indicate that crushing HS does not impact TPC. This is due to phenolic compounds being more abundant in the external part of the seeds (hull) than in the internal part (kernel) [48].

Foaming on the extract was observed during the extraction of DF seeds, after extraction and during the filtration step. This remarkable foaming ability after MA-SWE has been previously observed and attributed to the aggregation and disaggregation of proteins during the extraction process [31] and, alternatively, to potential interactions between proteins and polysaccharides, which are present in high concentrations in hemp seeds [10].

To conclude, it appears that a defatting step is a suitable pretreatment with which to enhance selectivity towards both phenolic compounds and proteins prior to MA-SWE. The recovery of oil during the defatting step, which is already considered valuable from an industrial standpoint, will ensure dual economic profit. The defatting step will thus guarantee the preparation of a nutritional and health-promoting extract, and a valuable oil, both of which have promising industrial applications [49]. For this reason, the following section will focus on the optimization of key extraction parameters during MA-SWE using DF seeds.

### 3.3. Box–Behnken-Based Optimization

To enhance the efficiency of the extraction process and increase the selectivity of targeted components in the final extract, the impact of extraction parameters (temperature, time and ratio) was investigated. The targeted components include the overall yield, polyphenol content, protein selectivity and recovery.

#### 3.3.1. Impact on the Yield

The yield is among the critical responses for evaluating the operationality and efficiency of extraction processes. As shown in Table 2, the extraction yield varied between 5.14 and 35.69 g extract/100 g DM. The highest yield was obtained at a temperature of 180 °C for a time of 60 min and using a ratio of 1:17.5, while the lowest yield was obtained at a temperature of 140 °C, at a ratio of 1:5 and for a time of 5 min. SWE has been demonstrated to yield higher results than conventional methods due to its efficacy in extracting a more extensive range of compounds of varying polarities [30].

**Figure 2:**
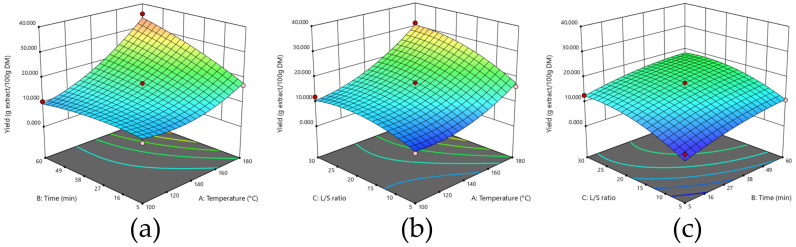
Effect of extraction variables on yield (g extract/100 g DM). (**a**) Time and temperature; (**b**) Temperature and ratio; and (**c**) ratio and time.

As shown in the 3D representations of the effects of the different interactions between variables on the extraction yield (Figure 2), combining high temperatures with longer process times and/or higher ratios was beneficial to the yield. The experimental data are represented by a second-order polynomial equation (Table 3), which fit well, with R^2^ = 0.9580. In the case of extraction yield, the most influential factor was temperature, which was approximately two times more impactful on yield than the other two factors. The analysis of variance ANOVA, represented in Table S1 in the Supplementary Materials, showed that all the linear effects were significant as well as two quadratic effects (A^2^ and C^2^) and one interaction (AB), all with a *p*-value under 0.05. The non-significant lack of fit (*p* > 0.05) for all the responses studied also confirms that the model adequately describes the experimental data (Table S1).

#### 3.3.2. Impact on the Protein Selectivity

Protein selectivity ranged between 27.30 and 45.83 g proteins/100 g extract, as shown in Table 2. HS proteins consist mainly of globulin and albumin, which are characterized by a remarkably high content of arginine and glutamic acid [13,14]. The lowest protein selectivity was observed in runs 5 and 9, with values of 27.38 g/100 g extract and 27.30 g/100 g extract, respectively, with these results being obtained using an extraction ratio of 1:5. In run 5, the conditions included a temperature of 100 °C and an extraction time of 32.5 min, whereas run 9 was conducted at a temperature of 140 °C and a shorter extraction time of 5 min. Meanwhile, the highest protein selectivity was achieved under conditions of 180 °C, an extraction time of 60 min and a ratio of 1:17.5. Elevated temperatures are well-documented to enhance protein-extraction efficiency [31].

**Figure 3:**
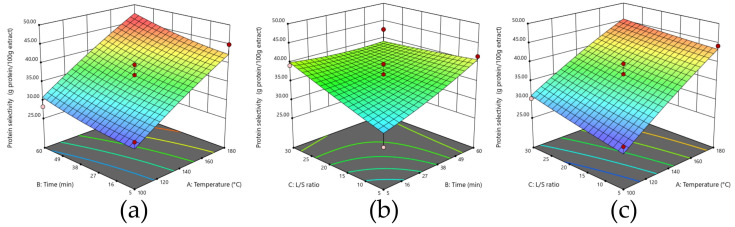
Effect of the extraction process variables on protein selectivity (g proteins/100 g extract): (**a**) time and temperature; (**b**) L/S ratio and time; (**c**) temperature and L/S ratio.

Figure 3 presents 3D representations of the responses of the different extraction conditions for the different runs. The second-order polynomial equation that represents the experimental data, presented in Table 3, exhibited an R^2^ = 0.8987. Of the variables, temperature emerged as the most influential factor, with an impact that is approximately four times greater than that of extraction time and ratio. ANOVA analyses, presented in Table S1 in Supplementary Materials, demonstrate that all linear effects were significant, as well as two quadratic effects (A^2^ and C^2^) and two interactions (AB and AC), with all *p*-values of under 0.05.

#### 3.3.3. Impact on the Protein Recovery

**Figure 4:**
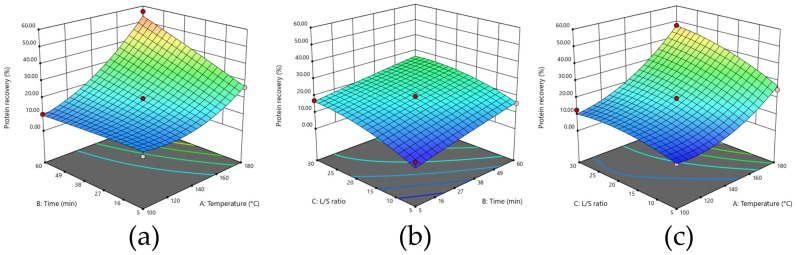
Effect of extraction process variables on protein recovery (%): (**a**) time and temperature; (**b**) L/S ratio and time; (**c**) temperature and L/S ratio.

The recovered protein results are reported in Table 2. As can be seen, the results throughout the runs fluctuated between 4.86 and 56.58%. The best result for protein recovery was found when using an extraction temperature of 180 °C for a time of 60 min at a ratio of 1:17.5. The highest recovery value was achieved in run 4 (56.58%), while the lowest value was obtained using a temperature of 140 °C for 5 min at with a ratio of 1:5 (run 9). These findings align with the observed trend for protein selectivity and highlight the promising potential of high temperatures, extended extraction time and relatively high ratios for efficient protein extraction from defatted HS. As shown in Figure 4, this combination consistently yielded favorable results.

As reported in Table 3, the second-order polynomial equation gave an R^2^ of 0.9809. From the equation, it was found that the factor with the most significant impact was temperature, with that impact being approximately 2.5 times higher than for the other factors. The ANOVA analysis of variance showed that all linear effects were significant as was one interaction (BC); all displayed *p*-values of under 0.05 (Table S1).

#### 3.3.4. Impact on the Recovery of Polyphenols

The TPC results, as revealed in Table 2, ranged between 2.06 and 6.57 g GAE/100 g of extract. The highest value was found in run 6, which was conducted with the following set of parameters: a temperature of 180 °C, an extraction time of 32.5 min and a ratio of 1:5. Furthermore, no statistical differences were noticed between runs 6 and 4 (6.57 and 6.54 g GAE/100 g extract), with both conducted at a temperature of 180 °C, but with run 4 having a longer extraction time (1 h) and a higher ratio (1:17.5). The lowest value was reached with run 5 (1.98 g GAE/100 g extract), conducted using a temperature of 100 °C for a time of 32.5 min and at a ratio of 1:5. However, the increased TPC at elevated temperatures does not strictly suggest the extraction of higher contents of phenolic compounds given the non-specific nature of the Folin–Ciocalteu assay, which may involve interferences from other reducing compounds [50]. However, similar outcomes have been reported by Ibañez et al., for the SWE of polyphenols from dried rosemary leaves at 100, 150 and 200 °C [51]. In conventional extraction methods, thermal degradation typically occurs at temperatures exceeding 80 °C [52]. Conversely, advanced extraction methods, such as SWE, employ a cell-based approach that prevents exposure to light and typically operates in an absence of oxygen (typically by purging the cell with nitrogen) for a comparatively brief period. This approach is often more efficient than traditional extraction methods, which often require prolonged operation. The prolonged exposure to light and oxygen during traditional extraction methods has been shown to degrade extractants [53]. A considerable body of literature has documented the increase in TPC during SWE at elevated temperatures [51,54], and this increase can be attributed to the release of insoluble phenolic compounds when the lignin bonds to phenolic acids are broken. Indeed, the dissociation of lignin–phenolic acid bonds or the decomposition of lignin, could lead to an increased concentration of phenolic acids [52].

**Figure 5:**
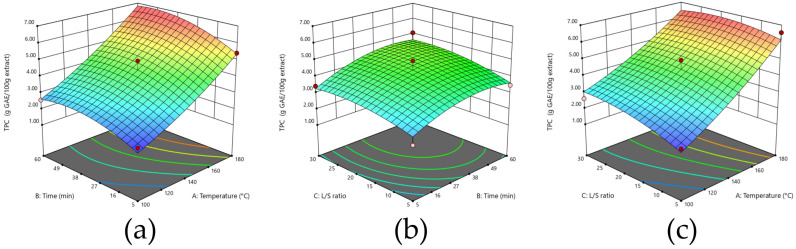
Effect of the extraction process variables on TPC (g GAE/100 g extract): (**a**) time and temperature; (**b**) L/S ratio and time; (**c**) temperature and L/S ratio.

Figure 5 highlights the existence of interesting interactions between a medium ratio and a medium extraction time and high temperatures. The degradation of more unstable compounds at elevated temperatures during prolonged extraction periods may serve as a possible explanation for this phenomenon [55]. On the other hand, the use of a medium ratio in the procedures is sufficient for the extraction of phenolic compounds, since these compounds are dominant in the external part of the seeds [41]. The experimental data are represented by a second polynomial equation, which can be found in Table 3, with a R^2^ = 0.9329, demonstrating a good fit. The equation revealed that temperature is the most influential factor, with an impact that is 3.5 to 5 times greater than those of the other variables.

The variance ANOVA analysis presented in Table S1 shows that all linear effects and one quadratic effect (B2) were significant, all with *p*-values below 0.05.

#### 3.3.5. Optimal MA-SWE Conditions

A multifaceted approach was employed to enhance the yield, protein selectivity, protein recovery and total phenolic content, with the implementation of a desirability function serving as the underlying optimization strategy. The initial set of optimal conditions, designated as “MAPPY” was obtained by varying all parameters across their full range to maximize the responses, leading to an optimal extraction temperature of 180 °C, an operative time of 57 min and a ratio of 1:28. A secondary set, designated “OEC,” was derived through the minimization of the parameters studied, while still achieving the best possible results. The optimal conditions in this case were identified to be: 165 °C, 35 min and a ratio of 1:16. These conditions were designed to minimize time, temperature and solvent volume, thereby reducing energy consumption in the MA-SWE process. The temperature was the most impactful independent variable for all responses, as shown in Table 3. This finding aligns with the observations reported in the study by Žagar et al. (2024) [30].

**Figure 6:**
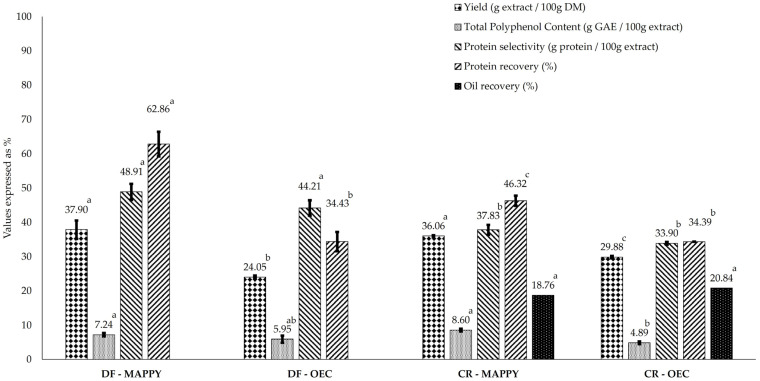
Impact of HS pretreatment (defatting and crushing) and optimal extraction conditions (MAPPY and OEC) on yield, protein selectivity, TPC and protein recovery. Distinct lowercase letters represent significant differences (*p* < 0.05).

The experimental and predicted response values of MA-SWE performed using “MAPPY” and “OEC” conditions are depicted in Table 4. These conditions were evaluated on both defatted and crushed non-defatted seeds and the results achieved, in terms of yield, protein selectivity, TPC and protein recovery, are shown in Figure 6.

The optimal conditions for extraction resulted in the highest yields across a range of parameters, suggesting their potential for maximizing MA-SWE efficiency. As can be seen in Table 4, the experimental results in terms of yield, protein recovery, TPC and protein selectivity were consistent with the predicted values, indicating that the model performed satisfactorily. Under “MAPPY” conditions, the highest protein content in the extracts obtained was 48.91 g/100 g extract. This largely exceeds the values reported by Švarc-Gajić and colleagues for the SWE of proteins from hemp cakes using different atmospheres, which range from 4.83 to 6.83 g per 100 g of extract [26]. However, the result is lower than the values obtained using the conventional pH-shift HS extraction method. Indeed, as reported in a study conducted by Potin et al. (2019), protein-recovery values that varied between 60 and 70% can be obtained at pH 12 [23], while protein selectivity above 96% was achieved in the research study conducted by M. Liu et al. (2023) [12]. Proteins obtained via the pH-shift extraction/isoelectric precipitation method have been shown to be off-flavor and exhibit a green color [56], which has limited their application in the food industry. The implementation of advanced technologies, such as SWE, may serve as a promising solution to address this challenge and maintain the protein’s native state. In the case of TPC, the values were around 7.24 g GAE/100 g extract. These findings demonstrate levels that exceed those documented in current literature. For instance, ultrasound-based extraction using acetone water at equal proportions yielded a maximum content of 5.37 g GAE/100 g extract [57], while conventional extraction with 75% ethanol, followed by ethyl acetate fractionation, yielded 5.67 g GAE/100 g extract [58].

Under OEC conditions, protein selectivity and TPC were determined to be 34.43 g/100 g extract and 5.95 g GAE/100 g extract, respectively. Despite exhibiting lower values than “MAPPY”, these conditions demonstrate significant potential in terms of sustainability. This is attributed to the reduced extraction time, ratio and temperature. Consequently, these conditions enhance the sustainability of the extraction process by minimizing both energy and water consumption.

When comparing the efficiency of these two optimal conditions on both defatted and crushed, non-defatted seeds (Figure 6), defatted seeds showed higher values, thus confirming the results obtained under the single, non-optimized conditions using different pretreated HS (Figure 1). These findings indicate that a defatting step is a suitable pretreatment with which to enhance selectivity towards both phenolic compounds and proteins prior to MA-SWE.

### 3.4. Functional Properties of the Final Products

**Figure 7:**
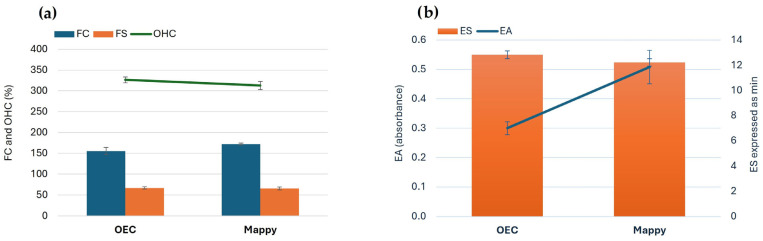
Functional properties of the optimized OEC and MAPPY extracts: (**a**) FC, FS and OHC, and (**b**) EA and ES. Abbreviations: EA: emulsification activity; ES: emulsification stability; FC: foaming capacity; FS: foaming stability; MAPPY: maximum achieved protein and polyphenol yield; OEC: optimal economic conditions; OHC: oil holding capacity. No statistically significant differences were found among the functional properties evaluated, with the exception of EA (*p* < 0.05).

The final products obtained using the two optimized conditions were subjected to analysis for their functional properties mainly linked to the protein fraction. As a matter of fact, food protein functionality is a matter of scale and scope, and must address a number of concerns including mainly performing its structural role, maintaining product quality and providing the desirable attribute [59]. However, in a more straightforward manner, protein functionality can refer to the way a protein behaves in the presence of other ingredients. Some of the main functional properties of proteins include solubility, organoleptic features (e.g., odor, color, flavor), viscosity, emulsification, foam formation, water absorption, lipid binding, among others [60]. The results achieved for the functional properties of the extracts obtained are reported in Figure 7.

As illustrated in Figure 7, a slightly higher OHC was obtained under “OEC” conditions (326.50% compared to 312.89% in the “MAPPY” sample, with a *p*-value > 0.05). This value can be attributed to the effect of the high content of phenolic compounds on the extracts obtained under “MAPPY” conditions. Indeed, maximizing polyphenols together with proteins can change protein structure and the whole extract structure at micro levels (via protein–polyphenol complexation or conjugation), thus reducing the availability of hydrophobic binding sites and/or reducing extract porosity/solubility [61]. Moderate protein-polyphenol binding, as may occur in the “OEC” extract due to its lower protein and TPC compared with “MAPPY”, can enhance oil retention, whereas excessive crosslinking may lead to a reduced OHC [62]. These results are within the same range of a previously published study on *Nigella sativa* protein isolate [63] and higher than those reported in others [64,65].

Regarding the FC, the average value of the “MAPPY” extract (172.25%) was slightly higher than that of “OEC” (155.51%) (*p*-value > 0.05). However, the FS was quite similar between both extracts (66.98 and 65.64% for the “OEC” and “MAPPY” extracts, respectively, with a *p*-value > 0.05). EA of the samples obtained under the “MAPPY” conditions was significantly higher (0.51) than the value obtained for the OEC sample (0.30) (*p*-value < 0.05), while no significant difference (*p*-value > 0.05) was found for the ES values (12.23 and 12.83 min, respectively). These results are comparable to those obtained for de-oiled soy flours and their protein isolates [65]. In another relevant study, a similar trend was observed for EA and ES as no significant differences were found for ES, while EA values were enhanced after thermal treatments [66].

The higher FC and EA observed for “MAPPY” may stem from its higher protein yield and the partial unfolding of proteins during its more intensive extraction process that aimed at maximizing phenolic and protein recovery. This might be related to the promotion of a balance of hydrophobic-hydrophilic properties of the extract, which has the potential to enhance emulsification and foaming properties [60]. The MAPPY process, optimized for maximum polyphenol and protein yields, could also have led to greater phenolic–protein complexation, which can alter surface hydrophobicity [67]. Another possible explanation for the higher EA in the “MAPPY” extract may be attributed to protein aggregations (protein-to-protein or protein-to-carbohydrate) upon the elevated thermal treatment under the “MAPPY” conditions, as evidenced by Ma and colleagues [66].

At the microstructural level, the thermo-mechanical conditions of the MA-SWE applied in this study can lead to protein denaturation and potentially generate process-induced compounds. For instance, controlled heating might be useful to modify the structure of food protein, thus leading to an improved functional property [60]. Likewise, the application of high-pressure can also lead to structural changes in protein, thus changing its functionality through the modification of its secondary, tertiary, and quaternary structures [60,68].

Hence, to better elucidate these structural modifications through the extraction process, an in-depth proteomics-based investigation would be of particular interest in profiling the proteome of the two optimized extracts and identifying structure-function relationships relevant for application. A comprehensive omics approach, integrating both proteomics and metabolomics, would further provide detailed insight into the molecular composition of the extracts, the interactions occurring between their bioactive and functional components, and any potential safety considerations for use in food formulations.

## 4. Conclusions

An advanced MA-SWE-based approach has been optimized using Box–Behnken design and applied for the simultaneous recovery of proteins and phenolic compounds from HS. The optimization of parameters to achieve the highest yield resulted in an extraction temperature of 180 °C, an operative time of 57 min and a ratio of 1:28. Optimization to identify the most sustainable experimental conditions with the aim of minimizing water and energy consumption provided the following parameters: 165 °C, 35 min and a ratio of 1:16. The experimental results in terms of yield, protein recovery, TPC and protein selectivity were consistent with the predicted values, indicating that the model performed satisfactorily. Considering the functional properties of the extracts obtained, both extraction strategies produced functionally comparable extracts. However, the higher foaming and emulsifying capacities observed in the MAPPY extracts suggest that more intensive extraction promoted partial protein unfolding and phenolic–protein complexation. Considering this, the findings demonstrate that a defatting step followed by MA-SWE is an effective strategy for recovering both phenolic compounds and proteins. Further studies are needed to evaluate the impact of these extraction conditions on the functional and health-promoting effects of these extracts in real-world applications. Additionally, future scale-up of the process is crucial for evaluating its feasibility and efficiency on an industrial scale.

## Figures and Tables

**Table 1:** Independent variables used in the extraction process and their attributed codes.

Variables	Unit	Symbol	Levels		
			1	0	−1
Temperature	°C	A	180	140	100
Time	min	B	60	32.5	5
Solid/liquid ratio	g/g	C	1:30	1:17.5	1:5

**Table 2:** Experimental variables and the responses obtained for the Box–Behnken design.

Run	Independent Variables			Responses			
	Temperature (°C)	Time (min)	S/L Ratio (g/g)	Yield (g Extract/100 g DM)	Protein Selectivity (g Proteins/100 g Extract)	Protein Recovery (%)	TPC (g GAE/100 g Extract)
1	100	5	17.5	9.58	28.56	9.47	2.06
2	180	5	17.5	16.97	44.94	26.37	5.41
3	100	60	17.5	10.38	28.26	10.15	2.56
4	180	60	17.5	35.69	45.83	56.58	6.54
5	100	32.5	5	5.63	27.38	5.33	1.98
6	180	32.5	5	16.25	44.26	24.88	6.57
7	100	32.5	30	12.06	30.30	12.63	2.61
8	180	32.5	30	31.41	43.75	47.53	6.28
9	140	5	5	5.14	27.30	4.86	2.21
10	140	60	5	10.86	41.58	15.62	3.48
11	140	5	30	12.71	39.20	17.23	3.42
12	140	60	30	15.98	42.49	23.48	5.07
13	140	32.5	17.5	15.37	35.28	18.76	4.19
14	140	32.5	17.5	14.00	35.51	17.20	4.29
15	140	32.5	17.5	15.43	36.88	19.69	4.16
16	140	32.5	17.5	17.85	39.61	19.90	4.95

**Table 3:** Designed equation models of the relationship between the variables used.

Responses	Equation	R^2^	R^2^ Adjusted
Yield	Y = 15 + 7.83A+ 3.56B + 4.28C + 4.48AB + 2.18AC + 3.83A^2^ − 3.16C^2^	0.9580	0.9213
TPC	Y = 4.38 + 1.95A + 0.5686B + 0.3918C − 0.5338B^2^	0.9329	0.9085
Protein selectivity	Y = 36.95 + 8.04A + 2.27B + 1.90C − 2.75BC	0.8987	0.8619
Protein recovery	Y = 18.75 + 14.72A + 5.99B + 6.28C + 7.38AB + 3.84AC + 7.02A^2^ − 3.32C^2^	0.9809	0.9641

**Table 4:** Predicted and experimental values in terms of yield, protein recovery, protein selectivity and TPC for both “MAPPY” and “OEC” conditions.

Conditions	Yield (g Extract/100 g DM)			Protein Recovery (%)			Protein Selectivity (g Proteins/100 g Extract)			TPC (g GAE/100 g Extract)		
	Predicted	Experimental	RSE	Predicted	Experimental	RSE	Predicted	Experimental	RSE	Predicted	Experimental	RSE
“MAPPY” (180 °C, 57 min, 1:28)	37.20	37.90 ± 2.66	1.88	58.90	62.86 ± 3.58	6.72	46.50	48.91 ± 2.34	5.18	6.70	7.24 ± 0.48	8.06
“OEC” (165 °C, 35 min, 1:16)	21.70	24.05 ± 0.48	9.77	31.30	34.43 ± 2.79	10.00	42.20	44.21 ± 2.23	4.76	5.60	5.95 ± 1.05	6.25

RSE: relative standard error expressed as percentage.

## Data Availability

Data will be made available upon request from the corresponding authors.

## Supplementary Materials

The following supporting information can be downloaded at https://www.mdpi.com/article/10.3390/foods14244201/s1, Table S1: Fitted models obtained from the analysis of variance (ANOVA); Figure S1. Calibration curve obtained for the analysis of total phenolic content (TPC).
