# Supplementary material for: Microwave-Assisted Subcritical Water Extraction of Hemp Seeds for the Simultaneous Recovery of Proteins and Phenolic Compounds
Source: Foods. 2025 Dec 7;14(24):4201. doi: 10.3390/foods14244201 (PMC12733026; doi:10.3390/foods14244201)
Supplement: Supplementary file 1 [file foods-14-04201-s001.zip › foods-3992585-supplementary.pdf]

Microwave-Assisted Subcritical Water Extraction of Hemp Seeds for the Simultaneous Recovery of Proteins and Phenolic Compounds

Aziadé Chemat [1,2], Salah Chaji [1], Christian Cravotto [3], Giorgio Capaldi [1], Luisa Boffa [1], Giorgio Grillo [1], Anne-Sylvie Fabiano-Tixier [2,*] and Giancarlo Cravotto [1,*]

[1] Department of Drug Science and Technology, University of Turin, 10125 Turin, Italy; aziade.chemat@gmail.com (A.C.); salah.chaji@unito.it (S.C.); giorgio.capaldi@unito.it (G.C.); luisa.boffa@unito.it (L.B.); giorgio.grillo@unito.it (G.G.)

[2] GREEN Extraction Team, National Research Institute for Agriculture, Food and Environment (INRAE), Mixed Research Unit (UMR) 408, Avignon University, 84000 Avignon, France

[3] Unité de Recherche et de Développement (URD) Agro-Biotechnologies Industrielles (ABI), Centre Européen de Biotechnologie et de Bioéconomie (CEBB), AgroParisTech, 51110 Pomacle, France;
christian.cravotto@agroparistech.fr

* Correspondence: anne-sylvie.fabiano@univ-avignon.fr (A.-S.F.-T.); giancarlo.cravotto@unito.it (G.C.)

Supplementary materials

Table S1. Fitted models obtained from the analysis of variance (ANOVA).

	Yield (g extract / 100g DM)		Protein recovery (%)		Protein selectivity (g protein / 100g extract)		TPC (g GAE/100g extract)	
	SS	*p*-value	SS	*p*-value	SS	*p*-value	SS	*p*-value
Model	937.14	<0.0001	2854.31	<0.0001	616.95	<0.0001	35.35	<0.0001
A - Temperature	490.91	<0.0001	1734.19	<0.0001	516.59	<0.0001	30.39	<0.0001
B – Time	101.55	0.0021	286.77	0.0002	41.26	0.0268	2.59	0.0065
C – Ratio	146.87	0.0007	315.04	0.0001	28.91	0.0557	1.23	0.0417
AB	80.35	0.0042	217.99	0.0005				
AC	19.04	0.0902	58.86	0.0196				
BC					30.19	0.0514		
A^2	58.56	0.0098	197.36	0.0007				
B^2							1.14	0.0484
C^2	39.86	0.0237	44.09	0.0360				
Residual	41.04		55.67		69.53		2.54	
Lack of fit	33.37	0.2299	51.13	0.0734	57.66	0.3372	2.13	0.3213
Pure error	7.67		4.54		11.87		0.4168	
Core total	978.18		2909.98		686.48		37.89	

2025
Chaji, S.;
G.; Boffa, L.;
/xxxxx
open access
terms and
Attribution (CC BY) license (https://creativecommons.org/licenses/by/4.0/).

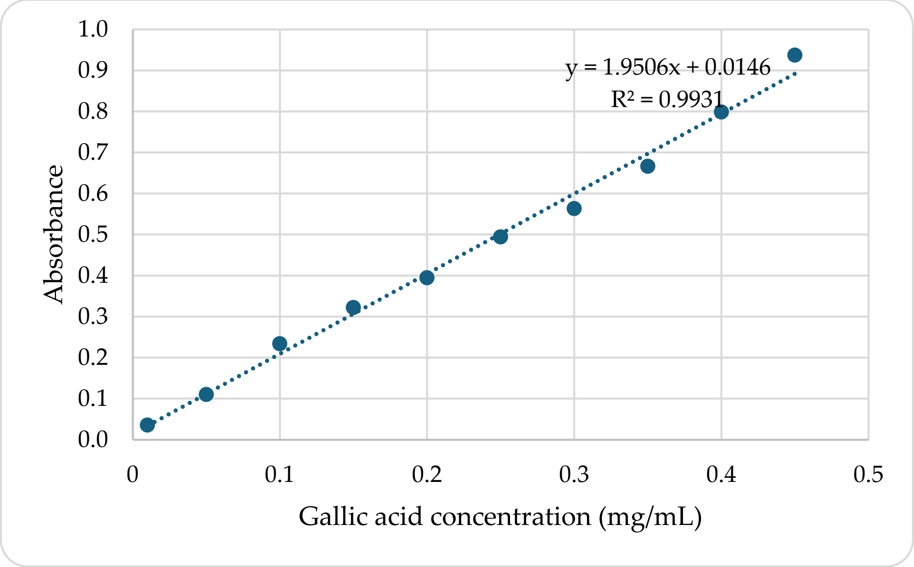

Figure S1. Calibration curve obtained for the analysis of total phenolic content (TPC).
